# A clinical pilot study to evaluate the correlation between pulse wave velocity and cardiac output during elective surgery

**DOI:** 10.1186/cc9480

**Published:** 2011-03-11

**Authors:** D Cain, S Harris

**Affiliations:** 1 University College Hospital, London, UK

## Introduction

Pulse wave velocity (PWV) is defined as the speed of conduction of a pressure wave generated by cardiac systole through the arterial tree. It may be non-invasively estimated from the pulse transit time that is measured as the interval between the R wave on an electrocardiogram and first inflexion point of the paired plethysmographic wave recorded from a finger pulse oximeter [1]. Within a simple two-component Windkessel model of the arterial system, PWV is proportional to the square root of arterial elastance [2]. Elastance is defined as the ratio of pulse pressure (PP) to stroke volume (SV). PWV might therefore provide a non-invasive estimate of cardiac output.

## Methods

Adult patients undergoing major elective surgery were eligible. PWV was recorded using HypnoPTT (Nelco Puritan-Bennet). Invasive arterial blood pressure measurements were preferred when available. Stroke volume was measured via ODM (Deltex Ohmeda). Values were recorded every 5 minutes, smoothed (median of five consecutive values) and converted to the centimetre gram second system. SV was derived from PWV: SVPWV PWV^2 ^× PP^-1^.

## Results

**Figure 1:**
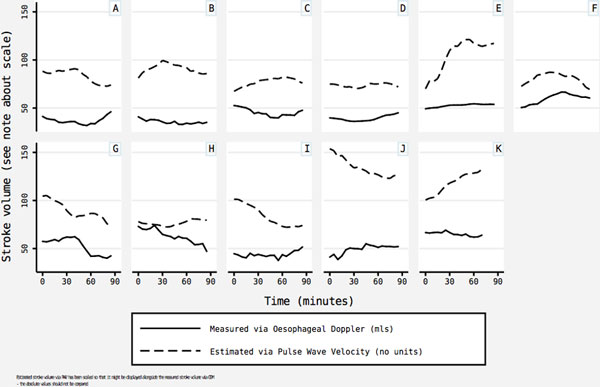
**Stroke volume during the first 90 minutes of surgery**.

Eleven patients (aged 45 to 74 years; five men, six women) were enrolled. Data are presented as (mean, SD). PWV was successfully measured on 287 occasions (324 cm/second, 48.5). SVPWV was calculated (62.4 ml, 18.3). SVODM values were (88.7, 4.86). Individual plots of SVPWV and paired SVODM were generated for each patient (Figure 1).

## Conclusions

Estimated SVPWV values were within a clinically expected range; however, visual inspection of the plots demonstrated no relationship between SVPWV and gold standard SVODM. Furthermore there was no relationship between raw PWV data and SVODM. It is possible that PWV recordings were unreliable. The limited range of SVODM will have compressed our data, making any relationship less evident. We conclude SVPWV is not an accurate estimate of SVODM.
